# Patients’ attitude towards bedside teaching in Tunisia

**DOI:** 10.5116/ijme.5669.ea24

**Published:** 2015-12-25

**Authors:** Arwa Ben Salah, Sana El Mhamdi, Ines Bouanene, Asma Sriha, Mohamed Soltani

**Affiliations:** 1 Department of Epidemiology and preventive medicine, University Hospital of Monastir, Tunisia

**Keywords:** Bedside teaching, medical education, medical students, patient acceptance of health care, Tunisia

## Abstract

**Objectives:**

To assess patient' reaction towards bedside teaching at the University Hospital of Monastir (Tunisia) and to identify the factors that may influence it.

**Methods:**

A cross-sectional study was conducted during December 2012 at the University Hospital of Monastir. Each department, except the psychiatric department and the intensive care units, was visited in one day. All inpatients present on the day of the study were interviewed by four trained female nurses using a structured questionnaire.

**Results:**

Of the 401 patients approached, 356 (88.8%) agreed to participate. In general, the results demonstrate that patients were positive toward medical students’ participation. The highest acceptance rates were found in situations where there is no direct contact between the patient and the student (e.g. when reading their medical file, attending ward rounds and observing doctor examining them). As the degree of students’ involvement increased, the refusal rate increased. Gender, age, educational level, marital status and the extent of students’ involvement in patient’s care were identified as the main factors affecting patients’ attitude. Conclusion: Taking advantage of this attitude, valorizing patient role as educator and using further learning methods in situations where patient’s consent for student involvement was not obtained should be considered to guarantee optimal care and safety to patients and good medical education to future physicians.

## Introduction

Bedside teaching is seen as one of the most important component of medical education. It provides students and trainees with an opportunity to learn several clinical skills such as history taking, physical examination, clinical reasoning, decision making, communication and professionalism.^1^ It requires considerable enthusiasm and commitment on the part of both teacher and learner and willingness to cooperate on the part of patient who plays a crucial role in this educational method.

Several studies have shown that the majority of patients had positive attitudes towards the involvement of medical students,^2^^-^^5^ they even enjoyed their contribution in improving the training of the medical workforce, resulting in improved healthcare for the whole population.^3^^,^^4^^,^^6^^,^^7^   However, these attitudes vary across regions and countries and seem to be determined by various socio-demographic factors and cultural issues.^3^Many of these factors, as female gender,^8^^-^^10^ male student’s gender,^11^ and Islam religion^11^^, ^^12^ have shown to be associated with greater refusal rates of medical students presence, which may hinder bedside teaching and even affect the quality of medical education, especially in countries, like Tunisia, where alternative learning approaches are not developed yet.

In fact, medical education in Tunisia is characterised by being very traditional, with large number of students and hospital based education. Clinical teaching takes place in the third to fifth years of medical training. During these three years, medical students rotate through different medical and surgical departments where they spend about four hours a day experiencing patient-based clinical teaching; some of them perform clinical examination on their own, later they report their findings to the supervising clinician who corrects or confirms these findings and demonstrates the correct examination.

Playing a passive role in this approach, patients simply act as “teaching material”. However, since the transition, patient’s rights and informed consent have gained greater visibility, and patients now have the right to choose whether to have medical students involved in their care or not. These facts have prompted us to conduct this study to assess Tunisian patients’ attitude towards bedside teaching at the University Hospital of Monastir and to identify factors that may potentially influence their decision to allow or refuse medical students’ participation in their care, so that academic institutions would be adequately prepared to address different scenarios in order to guarantee high quality medical education. The aims of this study were twofold. The first was to assess patients’ attitude towards the presence of medical students at the Teaching Hospital of Monastir. The second was to identify factors that may affect this attitude in order to act upon them for better bedside teaching.

## Methods

### Study design

We carried out a cross-sectional study in December 2012 at Fattouma Bourguiba University hospital of Monastir which is an 866 bed tertiary-level teaching hospital with 22 departments. Each department, except the psychiatric department and the intensive care units, was visited in one day to collect data.

### Study participants

All inpatients present, on the day of each department’s visit, were enrolled. For pediatrics participants (patients under 15 years old), we surveyed parents in order to determine their comfort with medical student involvement in the care of their children.

The consent of all patients was obtained after an explanation of the nature and the purpose of the study.  Also, the participants were assured the anonymity and the confidentiality of the collected information and that their participation would not affect the quality of care provided. For pediatrics participants (patients under 15 years old), the consent was obtained from their parents. All patients have the choice to whether or not they want to take part in this study. The study protocol and data collection instrument were reviewed and approved by the University Hospital of Monastir Ethics Board.

### Data collection method and procedure

Data were collected over a period of one month, December 2012, using a questionnaire designed on the basis of literature^8^ and piloted on a sample of 20 patients to ensure face validity and clarity. In view of the anticipated variance in participant literacy, the questionnaire was administered by four trained female nurses who were not part of the health care team.

The questionnaire contained 38 items under 3 main sections. Section I included demographic and socio-economic data (age, gender, nationality, marital status, educational level and occupation). Section II included 26 questions about patients’ acceptance regarding the involvement of medical students in care process. To each question, the participant had to choose between “permitting male students only”, “permitting female students only”, “permitting both genders of students” or “not to permit either gender of students”. The third section included three questions; a question about who did the patients think was involved in their care (student, doctors or both of them), a question about the manner that the presence of medical students in Teaching Hospitals did affect the quality of health care (it improves, doesn’t affect or worsens the quality of care) and a question about the level of patients’ satisfaction with the care given by students (very satisfied, satisfied, not satisfied or not satisfied at all).

### Statistical analysis

Data entry and analysis were performed using the Statistical Package for Social Sciences software (SPSS) version 18.0. We used Chi square test and fisher exact test to study the association between each item evaluating “patients’ reaction toward students” and socio-demographic variables (age, gender, marital status, educational level and occupation). For this analytical part, participants ‘answers were pooled in two modalities “to permit medical students regardless of their gender” and “not to permit medical students regardless of their gender” which corresponds to the answers “to permit male students only, to permit female students only and not to permit either genders”. Pediatric patients were excluded from univariate analysis. A p-value less than 0.05 was considered statistically significant.

## Results

### Study participants

Of the 401 interviewed patients, 356 agreed to participate (response rate of 88.8%). The mean age of all patients surveyed was 42.7 years (SD=21) and 59.8% of them (n=213) were female. The majority of the participants were medical patients (36.8%, 131) and surgical patients (31.7%, 113) while the rest were either obstetrics/gynecology patients (20.5%, 73) or pediatrics patients (11%, 39) (Table 1).

**Table 1:** Socio-demographic characteristics of patients in the teaching hospital of Monastir (Tunisia), December 2012 (n= 356)

Characteristics		N (%)
Age (years)		
	≤40	162(45.5)
	>40	194 (54.5)
Gender		
	Male	143(40.2)
	Female	213 (59.8)
Marital status		
	Married	281(78.9)
	Not Married	75 (21.1)
Educational level*		
	≤ Primary	196 (60.1)
	≥ Secondary	130 (39.9)
Occupation		
	Not employed	177 (44.8)
	Employed	179 (55.2)
Specialty/ Department		
	Medicine	131 (36.8)
	Surgery	113 (31.7)
	Pediatrics	39 (11)
	Obstetrics/Gynecology	73 (20.5)

*Question for patients aged more than 6 years (n= 326)

**Table 2:** Patients’ acceptance to medical student in teaching hospital of Monastir, December 2012

Items		Permit male students only n(%)	Permit female students only n(%)	Permit both genders of students n(%)	Not to permit either gender of students n(%)
To read their medical file		7(2)	2(0.5)	331(93)	16(4.5)
To be present in outpatient clinic		7(2)	3(0.8)	333(93.5)	13(3.7)
To attend the ward rounds		7(2)	2(0.6)	334(93.8)	13(3.7)
To be present in the operation theatre		7(2)	5(1.4)	321(90.2)	23(6.4)
To be present in the delivery room^*^		0(0)	37(18.7)	140(70.7)	21(10.6)
To take medical history	In the presence of a SD	7(2)	3(0.8)	332(93.3)	14(3.9)
	Without the presence of a SD	6(1.7)	3(0.8)	314(88.2)	33(9.3)
To perform chest auscultation	In the presence of a SD	1(0.3)	15(4.2)	322(90.4)	18(5.1)
	Without the presence of a SD	0(0)	19(5.3)	284(79.8)	53(14.9)
To perform breast exam^*^	In the presence of a SD	0(0)	38(19.2)	141(71.2)	19(9.6)
	Without the presence of a SD	0(0)	31(15.7)	125(63.1)	42(21.2)
To perform abdominal exam	In the presence of a SD	2(0.6)	23(6.4)	320(89.9)	11(3.1)
	Without the presence of a SD	4(1.1)	25(7)	283(79.5)	44(12.4)
Vaginal examination^*^	To perform	0(0)	50(25.3)	65(32.8)	83(41.9)
	To observe doctor performing it	1(0.5)	64(32.3)	92(46.5)	41(20.7)
Digital rectal examination^†^	To perform	6(1.8)	52(14.5)	143(40.1)	155 (43.6)
	To observe doctor performing it	6(1.8)	49(15.6)	126(39.7)	136 (42.9)
Repair of Episiotomy^*^	To perform	0(0)	42(21.2)	66(33.3)	90(45.5)
	To observe doctor performing it	0(0)	60(30.3)	96(48.5)	42(21.2)
Urinary catheterization	To perform	18(5.1)	47(13.2)	138(38.7)	153(43)
	To observe doctor performing it	15(4.2)	57(16)	214(60.1)	70(19.7)
To prescribe drugs		0(0)	1(0.3)	228(64)	127 (35.7)
To give drugs		0(0)	1(0.3)	284(79.8)	71(19.9)
To ensure follow-up visit in outpatient clinics		0(0)	2(0.6)	189(53.1)	165 (46.3)

*Questions for non pediatric female patients (n=198); †Question for non pediatric patients (n= 317); SD= Supervisor Doctor

**Table 3:** Patients' acceptance of medical students reading their medical files, being present in outpatient clinic, attending ward rounds and surgical intervention and taking medical history, in the teaching hospital of Monastir, December 2012 ( n= 317)

Variables		Read file		Be present in outpatient clinic		Attend ward round		Attend surgical intervention		Take medical history			
										With SD		Without SD	
		n(%)	*p*	n(%)	*p*	n(%)	*p*	n(%)	*p*	n(%)	*p*	n(%)	*p*
Gender			0.03		0.02		0.02		0.02		0.03		0.02
	Male	105(88.2)		105(88.2)		105(88.2)		100(84)		105(88.2)		99(83.2)	
	Female	188(94.9)		189(95.5)		189(95.5)		183(92.4)		188(94.9)		182(91.9)	
Age (years)			0.89		0.93		0.66		0.5		0.57		0.71
	≤ 40	114(92.7)		114(92.7)		113(91.9)		108(87.8)		115(93.5)		108(87.8)	
	>40	179(92.3)		180(92.8)		181(93.3)		175(90.2)		178(91.8)		173(89.2)	
Marital status			0.33		0.31		0.31		0.02		0.74		0.27
	Married	261(92.9)		262(93.2)		262(93.2)		255(90.7)		260(92.5)		251(89.3)	
	Single	32(88.9)		32(88.9)		32(88.9)		28(77.8)		33(91.7)		30(83.3)	
Educational level			0.92		0.78		0.87		0.67		0.74		0.9
	≤ Primary	174(92.6)		175(93.1)		174(92.6)		169(89.9)		173(92)		167(88.8)	
	≥ Secondary	119(92.2)		119(92.2)		120(93)		114(88.4)		120(93)		114(88.4)	
Professional status			0.53		0.66		0.66		0.66		0.14		0.91
	Unemployed	129(93.5)		129(93.5)		129(93.5)		122(88.4)		131(94.9)		122(88.4)	
	Employed	164(91.6)		165(92.2)		165(92.2)		161(89.9)		162(90.5)		159(88.8)	

**Table 4:** Patients' acceptance of medical students performing digital rectal examination, urethral catheterization and observing these procedures done on them, in the teaching hospital of Monastir, December 2012 (n=317)

Variables		Digital rectal examination				Urinary catheterization				To perform abdominal exam			
		To perform		To observe		To perform		To observe		With SD		Without SD	
		n(%)	*p*	n(%)	*p*	n(%)	*p*	n(%)	*p*	n(%)	*p*	n(%)	*p*
Gender			0.003		<10^-3^		0.004		<10^-3^		0.01		0.03
	Male	60(50.4)		86(72.3)		61(51.3)		89(74.8)		113(95)		102(85.7)	
	Female	66(33.3)		92(46.5)		69(34.8)		94(47.5)		169(85.4)		150(75.8)	
Age (years)			0.002		<10^-3^		0.001		<10^-3^		0.10		0.43
	≤ 40	36(29.3)		54(43.9)		36(29.3)		56(45.5)		105(85.4)		95(77.2)	
	>40	90(46.4)		124(63.9)		94(48.5)		127(65.5)		177(91.2)		157(80.9)	
Marital status			0.12		0.14		0.18		0.52		0.009		0.48
	Married	116(41.3)		162(57.7)		119(42.3)		164(58.4)		255(90.7)		225(80.1)	
	Single	10(27.8)		16(44.4)		11(30.6)		19(52.8)		27(75)		27(75)	
Educational level			0.02		0.14		0.006		0.13		0.52		0.87
	≤ Primary	85(45.2)		112(59.6)		89(47.3)		115(61.2)		169(89.9)		150(79.8)	
	≥Secondary	41(31.8)		66(51.2)		41(31.8)		68(52.7)		113(87.6)		102(79.1)	
Professional status			0.012		0.02		0.015		0.007		0.93		0.45
	Unemployed	44(31.9)		67(48.6)		46(33.3)		68(49.3)		123(89.1)		107(77.5)	
	Employed	82(45.8)		111(62)		84(46.9)		115(64.2)		159(88.8)		145(81)	

**Table 5:** Patients' acceptance of medical students prescribing drugs, giving them drugs and ensuring follow up visit in outpatient clinics, in the teaching hospital of Monastir, December 2012 ( n= 317)

Variables		To prescribe drugs		To give drugs		To ensure follow-up visit in outpatient clinics	
		n(%)	*p*	n(%)	*p*	n(%)	*p*
Gender			0.13		0.07		0.21
	Male	73(61.3)		88(73.9)		74(62.2)	
	Female	138(69.7)		163(82.3)		109(55.1)	
Age (years)			0.09		0.86		< 10^-3^
	≤ 40	75(61)		98(79.7)		51(41.5)	
	>40	136(70.1)		153(78.9)		132(68)	
Marital status			0.99		0.82		0.52
	Married	187(66.5)		223(79.4)		164(58.4)	
	Single	24(66.7)		28(77.8)		19(52.8)	
Educational level			0.15		0.37		0.008
	≤ Primary	131(69.7)		152(80.9)		120(63.8)	
	≥ Secondary	80 (62)		99(76.7)		63(48.8)	
Professional status			0.66		0.30		0.19
	Unemployed	90(65.2)		113(81.9)		74(53.6)	
	Employed	121(67.6)		138(77.1)		109(60.9)	

**Table 6:** Female patients' acceptance of medical students being present in the delivery room, performing breast exam and observing and performing vaginal exam and repair of episiotomy in the teaching hospital of Monastir, December 2012 (n=198)

Variables		being present in the delivery room		Vaginal examination				Repair of episiotomy				performing breast exam			
				To perform		To observe		To perform		To observe		With SD		Without SD	
		n(%)	*p*	n(%)	*p*	n(%)	*p*	n(%)	*p*	n(%)	*p*	n(%)	*p*	n(%)	*p*
Age (years)			0.002		0.02		0.009		0.03		0.01		0.001		0.03
	≤ 40	56(60.2)		23(24.7)		34(36.6)		24(25.8)		36(38.7)		56(60.2)		52(55.9)	
	>40	84(80)		42(40)		58(55.2)		42(40)		60(57.1)		85(81)		74(70.5)	
Marital status			0.002		0.006		0.001		0.005		<10^-3^		0.002		0.01
	Married	136(73.5)		65(35.1)		92(49.7)		66(35.7)		96(51.9)		137(74.1)		122(65.9)	
	Single	4(30.8)		0(0)		0(0)		0(0)		0(0)		4(30.8)		4(30.8)	
Educational level			0.17		0.27		0.44		0.12		0.34		0.006		0.17
	≤ Primary	87(74.4)		42(35.9)		57(48.7)		44(37.6)		60(51.3)		92(78.6)		79(67.5)	
	≥ Secondary	53(65.4)		23(28.4)		35(43.2)		22(27.2)		36(44.4)		49(60.5)		47(58)	
Professional status			0.69		0.46		0.33		0.76		0.43		0.59		0.89
	Unemployed	84(71.8)		36(30.8)		51(43.6)		38(32.5)		54(46.2)		85(72.6)		74(63.2)	
	Employed	56(69.1)		29(35.8)		41(50.6)		28(34.6)		42(51.9)		56(69.1)		52(64.2)	

### Patients’ reactions towards medical students

Table 2 summarizes the patients’ reactions towards the presence of medical students in Teaching Hospitals. Of the patients interviewed, 93% (n= 331) permit both male and female students to read their medical file. Also, the majority of the participants agreed to allow them to be present in the outpatient clinic during consultation (93.5%, 333), in the ward during ward rounds (93.8%, 334) and in the operation theatre during their surgical intervention (90.2%, 321).

When female patients were asked about their acceptance regarding the presence of medical students in the delivery room during childbirth, 10.6% of them (n= 21) refused male and female students, 18.7% (n=37) agreed only about female students while 70.7% (n=140) agreed about both genders of students to be present. Regarding clinical breast exam, 71.2% (n=141) approved to be examined by a medical student in the presence of a supervising doctor, while 63.1 % (n=125) approved the examination in the absence of a supervisor. The same applied to observing and performing procedures; more than 45% of women would permit both genders of students to observe doctor performing vaginal examination and repairing episiotomy, while only about 33% of them would accept that student perform these procedures (Table 2).

Several factors may explain patients’ reactions toward medical students. In fact, the patient’s gender was statistically associated to the acceptance of patients in allowing medical students to read their medical files (p-value 0.03), to be present in the outpatient clinic during consultation (p-value 0.02) and in the ward during ward rounds (p-value 0.02), to attend their surgical intervention (p-value 0.02) and to take their medical history with or without the presence of the doctor (p-value 0.03 and 0.02 respectively). Female patients were more likely to accept medical students, regardless of their gender, than male patients (Table 3).

The acceptance by patients to allow students to perform or observe some procedures being performed on them (as digital rectal examination and urinary catheterization) was found to be statically associated to patients’ gender, age and occupation (Table 4). Male patients, patients aged more than 40 years and employed person revealed higher acceptance to students compared to women, patient aged under 40 and unemployed patients.

Regarding the question of patient acceptance that students ensure follow-up visit in outpatient clinics, age and educational level were significantly associated to it (Table 5). Among female patients, a higher refusal rate was found among women aged under 40 years old and single women about accepting medical students to be present in the delivery room during childbirth (p-value 0.02), to perform clinical breast exam with or without the presence of the supervisor and to observe or perform vaginal exam and repair of episiotomy (Table 6). Table 6 shows also that educational level was statically associated to the allowance of trainees to perform breast exam in the presence of a supervising doctor.

When asked about who is involved in their care process, 78.1% (n=278) answered that only doctors were treating them, while 21.9% (n=78) thought that students were involved too.  In the question about the level of satisfaction by the care provided by medical students, 39% (n=139) of the patients answered that they were very satisfied, 54.2% (n=193) reported that they were satisfied and 6.8 % (n= 24) were either not satisfied or not satisfied at all. Most respondents (79.5%, 283) thought that the involvement of medical students improves the quality of health care, only 2% (n=7) thought that their presence worsens it and 18.8% (n=67) believed that there is no relationship between quality of care and students.

## Discussion

This study contributed to the understanding of Tunisian patients ‘attitude towards the involvement of medical students in their care.  It showed a high level of patients’ acceptability of the presence of medical students in Teaching Hospitals. This finding coincides with the results of other studies in the Arab World^8^^,^^13^^-^^16^ and in developed countries.^3^^,^^17^^,^^18^ Many possible reasons for this high allowance were discussed in the literature, as the patients’ desire to contribute to medical education, the extra time that physicians may spend with them, and the opportunity to talk about their problem and to learn more about their condition.^5^^, ^^17^^, ^^19^^-^^22^

This positive reaction varied with the extent of involvement of medical students in the care process. The highest levels of acceptability were found in situations where there is a minimal direct contact between patients and students; such as reading medical files (93%, 331), being present in outpatient clinic (93.5%, 333) and attending ward rounds (93.8%, 334).There was however, some reluctance to students’ presence during childbirth (70.7%, 140), pelvic examination and procedures (such as digital rectal examination, urethral catheterization, vaginal examination etc.). This finding has been documented in several studies especially those conducted in Muslim countries where cultural and religious issues might affect the attitude of patients toward medical students and particularly the attitude of female patients toward male students^5^^,^^13^^,^^14^^,^^23^ which may lead to a poorer clinical experience for male students.^24^

As the degree of student involvement increased (from observation to history taking to examination and procedures), the refusal rate increased; especially when the parts examined were obviously sensitive (such as vaginal examination and digital rectal examination). This may be due to privacy-related concerns. Another common reason for objecting towards students’ involvement in physical examination, reported in the literature, was low confidence in medical students ‘skills to do a proper examination that detects findings^8^^,^^24^ which may explain the fact that the refusal rate was lower when an exam was performed by the student in the clinician’s supervision. This finding is consistent with other studies^8^^,^^15^^,^^24^^,^^25^  and matches with the results of Sayed-Hassan RM et al,^5^ who concluded that the patient’s feeling of safety and comfort is correlated to the presence of a supervisor.

The study also revealed that patients ‘reaction towards medical students depends on certain characteristics of patients themselves (such as gender, age, marital status, education level and occupation). Female patients showed higher acceptance of both genders of medical students when asked about situations where there was a minimal direct contact with students. However, when significant disrobing and embarrassing examinations were performed (for example during digital rectal examination or urinary catheterization), they were less likely than male patients to accept students of either gender. This finding is in agreement with that previously obtained by Shah-Khan M et al,^10^ and by Shann S et al ^9^ and may be explained by the higher sensitivity of women compared to men. On the other hand, patients with positive reaction towards the involvement of students in pelvic examination and intimate procedures were likely to be older (≥ 40 years old) and to have lower educational level. This could be because older patients are less likely to get embarrassed when exposed in front of others, and patients who had lower educational level believed that they had not the right to refuse medical students. However, in the study of Anfinan et al,^14^ and that of Shah-Khan et al,^10^ no significant association was observed between patients‘ attitudes and their age or their educational level. The influence of marital status had been shown only among women patients when asked about their reaction towards students’ involvement in breast and pelvic examination and procedures, and may be explained by the fact that married women have an experience with gynecological examination.

Other studies have indicated that religion,^11^economic level,^5^^,^^26^ severity of diagnoses^10^ and previous experiences with medical trainees^2^^,^^3^^,^^27^^,^^28^ may affect patients’ reaction toward medical students. Besides, Saeed F et al^26^ has documented that informing patients about the presence of student and obtaining their consent was associated with a positive attitude to be involved in teaching. In this study, only 21.9% of participants (n=78) thought that students were involved in their care which means that the majority of patients were not informed about the presence of medical students and therefore their consent was not obtained. This finding is in agreement with the result of O'Flynn N et al^29^ who found that 28% of patients believed that they did not have a choice about students presence and participation. Moreover, Sayad-Hassan RM et al,^5^reported that more than two thirds of patients were unaware of their right to refuse or accept the involvement of medical student, which represents an ethical issue given the mandatory nature of patients’ consent and its crucial role in establishing a positive patient and medical student relationship.^13^ Indeed, the acceptance and willingness of patients to be involved in clinical teaching should not be taken for granted. Instead, we should seek methods to promote patients’ cooperation. Communication is one of the strategic pillars on which we have to focus; by informing patients of the presence of the student and explaining to them his role and the degree of his involvement, we can reduce their fear and convince them to accept to be involved in the teaching process. Targeted messages should be delivered especially to single young women, people under 40 years old and well educated patients who showed some reluctance to students’ involvement in physical examination and procedures. On the other hand, we should valorize patients’ role as educators; as they have unique expertise derived from their experience of illness, disability or the effects of the social determinants of health, important messages that cannot be taught to students from a textbook.^30^ In this context, an active involvement of patients as educators is increasingly being recognized as a powerful educational strategy for both patients and learners. Bleakley and Bligh^31^ build on these concepts to propose a radical overhaul of conventional doctor-led medical education leading to an authentic patient-centred model that shifts the locus of learning from the relationship between doctor (as teacher) and student (as learner) with patients playing a supportive role, to the relationship between patient (as educator) and student (both as learner and co-educator) with the doctor-educator playing a supportive role.

However, in situations where patients’ consent for students involvement may not always be obtained, alternative strategies should be sought to ensure that students develop the required competencies; simulation using manikins and models should be considered as a standard method for learning intimate examination and invasive procedures for both male and female students without harming, disturbing or embarrassing patients.^32^ Another valuable tool in the teaching of physical diagnosis is the use of simulating patients who can provide good training on the choreography of physical examination, increasing students’ confidence in their techniques. Finally, the use of interactive multimedia programs (live demonstrations, slide shows, computerized animations and videotapes), should be explored as possible tools to improve physical diagnosis skills.^1^^,^^33^ Although effective and safe, all these methods should be considered to address scenarios that most patients are unwilling to allow students to participate but not to replace bedside teaching.

### Limitations and strengths

The important strength of this study is its originality. This study is the first one in Tunisia that aims to assess the patients’ acceptability of medical students in a Teaching Hospital, in a country where medical education is based on bedside teaching .Social desirability bias may be a limitation to our study. In fact, patients were surveyed while still hospitalized which could have influenced response rate and answers. However, the questionnaire was administrated by nurses, who were not part of health care team members and who have informed patients that their answers would not affect the quality of care provided.

We must also emphasize that the number of patients involved in bedside teaching the day of the interview was unknown; nevertheless, we do not think that this can influence the results since all patients had experienced bedside teaching during their hospital stay. In fact, every day, all newly admitted patients undergo real case based teaching.

## Conclusions

In conclusion, Tunisian patients showed overall positive attitude towards bedside teaching. This attitude appeared to be affected by patients’ characteristics (gender, age, educational level and marital status) and the extent of students’ involvement in their care. Taking advantage of this attitude, valorizing patients’ role as educators and using further learning methods in situations where patients’ consent for student involvement was not obtained should be considered to guarantee optimal care and safety to patients and good medical education to future physicians.

### Acknowledgements

Authors would like to thank all teams in the University Hospital of Monastir for their commitment and help.

### Conflict of Interest

The authors declare that they have no conflict of interest.
